# Dek overexpression in murine epithelia increases overt esophageal squamous cell carcinoma incidence

**DOI:** 10.1371/journal.pgen.1007227

**Published:** 2018-03-14

**Authors:** Marie C. Matrka, Katherine A. Cimperman, Sarah R. Haas, Geraldine Guasch, Lisa A. Ehrman, Ronald R. Waclaw, Kakajan Komurov, Adam Lane, Kathryn A. Wikenheiser-Brokamp, Susanne I. Wells

**Affiliations:** 1 Division of Oncology, Cancer and Blood Diseases Institute, Cincinnati Children's Hospital Medical Center, Cincinnati, OH, United States of America; 2 Centre de Recherche en Cancérologie de Marseille (CRCM), Inserm, U1068, CNRS, UMR7258, Institute Paoli-Calmettes, Aix-Marseille University, Marseille, France; 3 Division of Experimental Hematology & Cancer Biology, Cincinnati Children's Hospital Medical Center, Cincinnati, OH, United States of America; 4 Division of Bone Marrow Transplant and Immune Deficiency, Cancer and Blood Diseases Institute, Cincinnati Children’s Hospital Medical Center, Cincinnati, OH, United States of America; 5 Division of Pathology & Laboratory Medicine and Perinatal Institute Division of Pulmonary Biology, Cincinnati Children's Hospital Medical Center and Department of Pathology & Laboratory Medicine, University of Cincinnati College of Medicine, Cincinnati, OH, United States of America; Department of Genetics, St. Jude Children's Research Hospital, Memphis, UNITED STATES

## Abstract

Esophageal cancer occurs as either squamous cell carcinoma (ESCC) or adenocarcinoma. ESCCs comprise almost 90% of cases worldwide, and recur with a less than 15% five-year survival rate despite available treatments. The identification of new ESCC drivers and therapeutic targets is critical for improving outcomes. Here we report that expression of the human DEK oncogene is strongly upregulated in esophageal SCC based on data in the cancer genome atlas (TCGA). DEK is a chromatin-associated protein with important roles in several nuclear processes including gene transcription, epigenetics, and DNA repair. Our previous data have utilized a murine knockout model to demonstrate that Dek expression is required for oral and esophageal SCC growth. Also, DEK overexpression in human keratinocytes, the cell of origin for SCC, was sufficient to cause hyperplasia in 3D organotypic raft cultures that mimic human skin, thus linking high DEK expression in keratinocytes to oncogenic phenotypes. However, the role of DEK over-expression in ESCC development remains unknown in human cells or genetic mouse models. To define the consequences of Dek overexpression *in vivo*, we generated and validated a tetracycline responsive *Dek* transgenic mouse model referred to as *Bi-L-Dek*. Dek overexpression was induced in the basal keratinocytes of stratified squamous epithelium by crossing *Bi-L-Dek* mice to keratin 5 tetracycline transactivator (*K5-tTA*) mice. Conditional transgene expression was validated in the resulting *Bi-L-Dek_K5-tTA* mice and was suppressed with doxycycline treatment in the tetracycline-off system. The mice were subjected to an established HNSCC and esophageal carcinogenesis protocol using the chemical carcinogen 4-nitroquinoline 1-oxide (4NQO). Dek overexpression stimulated gross esophageal tumor development, when compared to doxycycline treated control mice. Furthermore, high Dek expression caused a trend toward esophageal hyperplasia in 4NQO treated mice. Taken together, these data demonstrate that Dek overexpression in the cell of origin for SCC is sufficient to promote esophageal SCC development *in vivo*.

## Author summary

The DEK oncogene is overexpressed in nearly all human cancers and portends a poor prognosis for many cancer types. High DEK expression causes cancer related phenotypes such as increased cellular proliferation, migration, and invasion *in vitro*. Despite the well documented link between high DEK expression and cancer, the consequences of Dek overexpression *in vivo* are poorly understood. To determine if Dek contributes to carcinogenesis in vivo, we generated a Dek inducible transgenic mouse model wherein Dek can be overexpressed in specific tissues and inhibited with the drug doxycycline. We targeted Dek overexpression to keratinocytes, the cell of origin for squamous cell carcinoma, and exposed the mice to the chemical carcinogen 4NQO to induce oral cavity and esophageal carcinogenesis. We found that DEK overexpression was sufficient to increase gross esophageal squamous cell carcinoma incidence and caused a trend toward increased cellular proliferation in adjacent non-tumor tissue. Importantly, we demonstrate for the first time that Dek overexpression specifically targeted to basal keratinocytes is sufficient to promote cellular and squamous cell carcinoma growth *in vivo*.

## Introduction

The human DEK oncoprotein is a predominantly chromatin-bound factor that regulates nuclear processes such as chromatin architecture, epigenetics, transcription and DNA repair [1–18]. DEK was originally identified as a fusion protein with the CAN/NUP214 nucleoporin in a patient with acute myeloid leukemia harboring the chromosomal translocation (t6;9)(p23;q34) [19]. Since its discovery, DEK was also shown to be increased in acute myeloid leukemia types that do not harbor the DEK-NUP214 fusion protein [20–22] and to be frequently overexpressed in solid tumors including colon, breast, gastric adenocarcinoma, ovarian carcinomas, bladder cancer, retinoblastoma, lung, pancreatic, neuroendocrine prostate cancer, hepatocellular, skin cancer, head and neck cancer squamous cell carcinoma (HNSCC), and esophageal squamous cell carcinoma (ESCC; **S5 Fig**) [23–40]. Additionally, high DEK expression is associated with poor prognosis in melanoma, gastric, ovarian, breast, prostate, bladder, lung, pancreatic, skin cancer, and head and neck SCC [25, 26, 30, 31, 33–35, 40–43].

Esophageal carcinomas are the sixth most common cause of cancer related death worldwide, and eighth in incidence worldwide [44–46]. Esophageal carcinoma occurs as either SCC or adenocarcinoma [47]. Esophageal SCC accounts for one third of esophageal cancer cases in the United States but represents more than 90% cases of esophageal cancer worldwide [47, 48]. The most common risk factors for ESCCs, similar to HNSCC, include tobacco smoke, heavy alcohol consumption, and infection with human papillomavirus [49, 50]. Several studies have additionally revealed that ESCC and HNSCC harbor similar genetic and molecular alterations [44, 51–54] and are treated with similar regimen of surgery and chemoradiation [50]. However, the 5-year survival rate for patients with HNSCC is over 50%, while for patients with ESCC it remains at a dismal 5–15% [45, 46, 48]. Current treatment regimens frequently result in irreparable tissue damage and disfiguration that additionally highlight the need for continued identification of oncogenic drivers and targeted therapies [55]. SCC arises from keratinocytes in squamous epithelium, and the overexpression of DEK has been shown to promote cell survival, proliferation, and transformation in combination with classical oncogenes while inhibiting apoptosis, cellular differentiation and senescence [16, 56–59]. DEK overexpression occurs through various mechanisms including gene amplification, increased transcription, and mutations in microRNAs and ubiquitin ligases responsible for DEK mRNA and protein degradation, respectively [60–70]. Several *in vivo* studies demonstrate the critical role of human and murine Dek in driving benign and malignant tumor growth. For example, *Dek* knockout (*Dek-/-*) mice are partially resistant to the formation of benign skin papillomas when treated with DMBA and TPA, a tumor initiator and promoter, respectively [16]. In a breast cancer mouse model, *Dek-/-* mice bred to Ron receptor tyrosine kinase transgenic mice, displayed a delayed onset of mammary tumors compared to *Dek+/+* mice [71]. In another study, *Dek* knockout *(Dek-/-) HPV E7* oncogene transgenic mice were protected from 4-nitroquinoline 1-oxide (4NQO)-induced HNSCC and ESCC tumor growth, but not initiation, when compared to their *Dek+/+* counterparts [39]. Taken together, these studies support the possible importance of Dek overexpression as a key driver of uncontrolled cellular growth and tumor development.

Historically, most of the data that links DEK overexpression to oncogenic phenotypes were obtained from knockdown and knockout model systems. Only recently has DEK overexpression been investigated *in vivo*. In a 2017 report, Nakashima et. al. generated tetracycline inducible, whole body, Dek over-expressing mice [72]. The mice were treated with 4NQO in the drinking water for 28 weeks to induce oral lesions, then induced to overexpress Dek for 4 weeks before sacrifice. 4NQO is a chemical carcinogen that mimics the effects of tobacco smoke by forming DNA adducts and mutations similar to those seen in human HNSCC and ESCC [73, 74]. When administered in drinking water, 4NQO stimulates susceptibility to squamous cell carcinomas in the tongue, oral cavity, and esophagus [73, 75]. In the study, the mice over-expressing Dek for 4 weeks, post 4NQO treatment, harbored significantly increased hyperplasia in the tongue with a trend toward increased tongue tumor incidence. Interestingly, Dek overexpression significantly decreased tongue tumor diameter [72]. This suggests that a short term induction of Dek overexpression after long term carcinogen exposure has pro- and anti-tumorigenic effects. Importantly, this study demonstrated that Dek overexpression promotes cellular proliferation in tissues exposed to carcinogens. However, whether these effects are due to high Dek expression in keratinocytes as the cell of origin, and/or other cell types, remains unknown. Therefore, we targeted long term induction of the Dek transgene to the stratified squamous epithelium, and monitored resulting tumor phenotypes.

To this end, a tetracycline responsive *Dek* and *luciferase* transgenic *Bi-L-Dek* mouse model was newly generated. *Bi-L-Dek* transgenic mice harbor a tetracycline response element (TRE) that controls the bi-directional expression of Dek and firefly luciferase. The TRE allows for temporal and tissue specific control of Dek overexpression, thus making it a versatile mouse model wherein the Dek transgene expression is controlled by tetracycline or its more stable derivative, doxycycline (dox). *Bi-L-Dek* mice were crossed to keratin 5 tetracycline transactivator *(K5-tTA*) transgenic mice to target Dek and luciferase expression to basal keratinocytes that serve as progenitor cells for stratified squamous epithelium and are the cell of origin for squamous cell carcinoma (SCC) of the tongue and esophagus. The tTA protein produces a tet-off system where expression of the Dek transgene is repressed by dox. Dek overexpression and transgene repression by dox was verified in the skin, tongue and esophagus. Once validated, *Bi-L-Dek_K5-tTA* mice were subjected to 4NQO treatment in the presence or absence of dox. Dek caused a trend toward increased proliferation in tongue and esophageal epithelium after 4NQO treatment. Furthermore, Dek overexpression was sufficient to increase the incidence of gross esophageal, but not oral, SCC tumor formation in this system. This data suggests that Dek contributes to ESCC tumorigenesis at least partially through keratinocyte intrinsic pathways which promote cellular and tumor growth.

## Results

### Generation of a conditional *Dek* transgenic tetracycline-off mouse model

**Fig 1:**
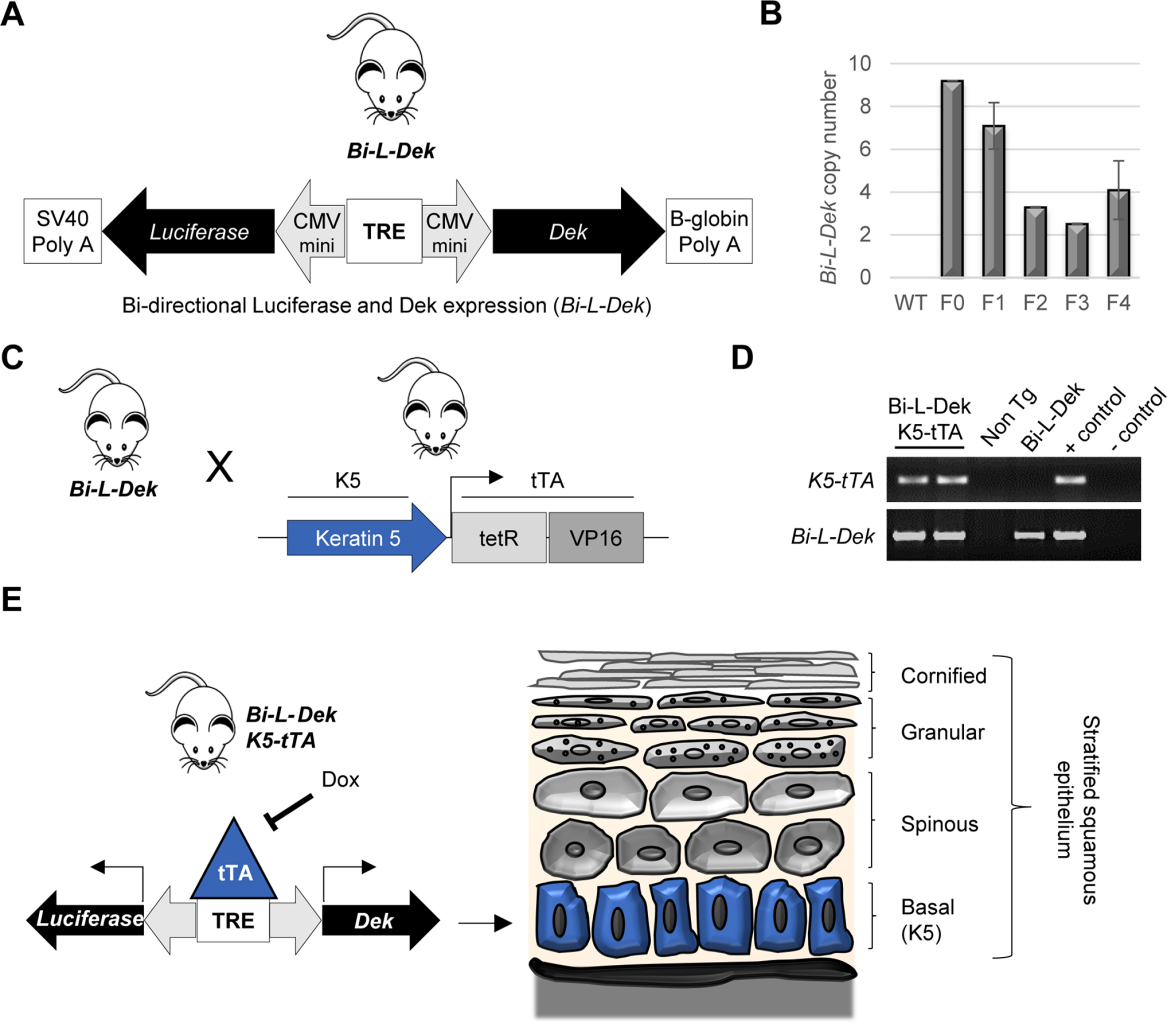
Generation of a tetracycline off *Dek* transgenic mouse model. (**A**) *Bi-L-Dek* transgenic mice were engineered by micronuclear injection of linearized *Bi-L-Dek* DNA into the pronucleus of FVB/N fertilized eggs. *Bi-L-Dek* mice harbor a tetracycline response element (TRE) that controls two mini cytomegalovirus (CMV) promoters driving bi-directional transcription of *Dek* and *luciferase*. (**B**) Copy number analysis of the *Bi-L-Dek* transgene in founder #317 identified 2–4 insertions in the F2-F4 generation. Error bars represent differences between 2–3 mice for each generation excluding F0 for which only one mouse exists. F3 and subsequent generations from this founder line were used for the experiments. **(C)** *Bi-L-Dek* mice were bred to keratin 5 promoter driven tetracycline transactivator (*K5-tTA)* mice. (**D**) *Bi-L-Dek* and *K5-tTA* transgene presence in offspring was confirmed by genotyping along with identification of single transgenic and non-transgenic (Non Tg) littermates. FVB/N (WT) mice were negative controls (-) and the F2 parent carrying the transgene was the positive control (+). (**E**) Schematic of *Bi-L-Dek_K5-tTA* mice designed to express luciferase and to overexpress Dek in the K5-positive basal layer of stratified squamous epithelium (highlighted in blue). Transgene repression by dox in this tet-off system is indicated.

In order to overexpress Dek conditionally in a tissue specific manner, we utilized a construct *Bi-L-Dek* wherein Dek and luciferase gene expression were driven by a tetracycline response element (TRE). To generate the *Bi-L-Dek* transgene, Dek cDNA was cloned into the Bi-L-Tet plasmid expression vector (Clontech, Mountain View, CA, USA) as published by others [76], described in the Materials and Methods, and illustrated in **S1A Fig**. Following validation of TRE-dependent Dek and luciferase expression in vector transfected cells **(S1B–S1E Fig)**, the transgene was excised and injected into the pronucleus of fertilized mouse eggs for generation of *Bi-L-Dek* transgenic founders (**S1F Fig**). *Bi-L-Dek* mice harbor a TRE that controls two mini cytomegalovirus (CMV) promoters driving bi-directional transcription of *Dek* and *luciferase* (**Fig 1A**). Four *Bi-L-Dek* transgenic founder lines were assessed for transgene stability over four generations before screening for doxycycline responsive expression of Dek (**Fig 1B**; the data for founder line #317 used in subsequent experiments is shown).

To determine which lines expressed Dek and luciferase under control of the TRE, *Bi-L-Dek* mice were crossed to *K5-tTA* mice (**Fig 1C and 1D**). The keratin 5 promoter targets tTA protein expression to the basal layer of stratified squamous epithelium including that of the esophagus, tongue, and skin (**Fig 1E**). In this system, administration of doxycycline (dox) represses tTA binding to the TRE to inhibit *Bi-L-Dek* transgene expression (**Fig 1E**). Founder #317 was chosen for subsequent experiments, harbors approximately three copies of the transgene (**Fig 1B**), and is referred to as *Bi-L-Dek* from here on.

### *Bi-L-Dek_K5-tTA* mice overexpress Dek conditionally in stratified squamous epithelium

**Fig 2:**
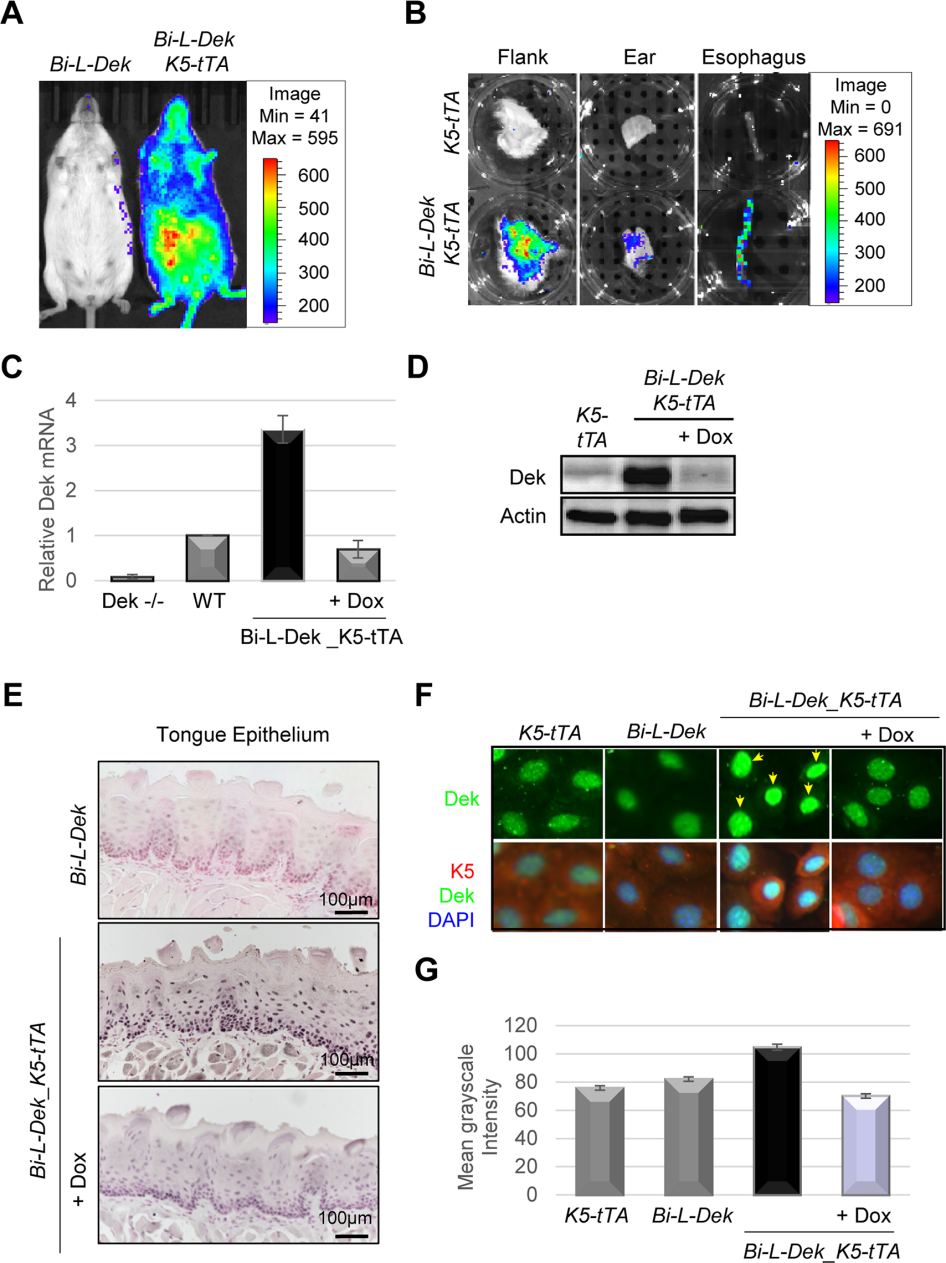
*Bi-L-Dek_K5-tTA* mice express luciferase and overexpress Dek in stratified squamous epithelium. (**A**) *In vivo* imaging system (IVIS) analysis depicts a single (*Bi-L-Dek*) and a bi-transgenic (*Bi-L-Dek_K5-tTA)* mouse after intraperitoneal injection of luciferin for luciferase detection in the skin of *Bi-L-Dek_K5-tTA* mice. (**B**) *Ex vivo* IVIS analysis of single transgenic (*K5-tTA*) versus bi-transgenic *(Bi-L-Dek_K5-tTA)* flank skin, ear, and esophagus following injection of luciferin, sacrifice, and dissection. (**C**) RT- qPCR of Dek mRNA levels in skin epithelium obtained from the flank of mice show a 3 fold induction of Dek transcript levels that is repressed to endogenous levels after seven days on dox chow. Primers detect endogenous and exogenous *Dek*. Error bars represent three mice for each genotype excluding the *Dek-/-* negative control which represents one mouse repeated in triplicate. (**D**) Representative western blot analysis for the detection of Dek protein levels in flank skin epithelium demonstrates increased levels of Dek protein in *Bi-L-Dek_K5-tTA* mice over those on dox and single transgenic controls. (**E**) Immunohistochemistry (IHC) with DEK antibodies (BD Biosciences, San Jose, CA, USA) in tongue epithelium confirms Dek protein overexpression in *Bi-L-Dek_K5-tTA* mice that is repressed within seven days of dox chow. (**F**) Immunofluorescence (IF) of cultured skin keratinocytes isolated from newborn *Bi-L-Dek_K5-tTA* pups with or without dox and their single transgenic littermates. Dox treated keratinocytes were cultured with 1ug/ml of dox for 48 hours before fixation. IF images of keratinocytes were taken at the same magnification and exposure after being probed for Dek, keratin 5 (K5), and stained with DAPI. (**G**) The mean fluorescent intensity of Dek staining in **2F** was quantified using ImageJ software (National Institutes of Health, Bethesda, Maryland, USA) [89].

*Bi-L-Dek* transgene expression in *Bi-L-Dek_K5-tTA* mice was validated with multiple methodologies. These included an *in vivo* imaging system (IVIS), and the detection of Dek mRNA and protein expression by quantitative, real time polymerase chain reaction (RT-qPCR), western blot analysis, *in situ* immunohistochemistry (IHC) and immunofluorescence (IF) **(Fig 2)**. IVIS and *ex vivo* imaging confirmed luciferase expression in the skin of *Bi-L-Dek_K5-tTA* bi-transgenic mice and in the esophagus (**Fig 2A and 2B**). Dek mRNA levels were induced 3.5 fold over endogenous levels in the skin of *Bi-L-Dek_K5-tTA* mice, and repression to endogenous levels was achieved by feeding with dox chow for seven days (**Fig 2C**). Dek protein expression in the skin also increased approximately 3 fold over the levels of endogenous Dek in the *Bi-L-Dek_K5-tTA* mice (**Fig 2D**). Dek overexpression in the tongue was detected by IHC along with the expected decrease in the corresponding mice on dox chow (**Fig 2E**). Finally, we isolated keratinocytes from *Bi-L-Dek_K5-tTA* skin for cell culture and performed IF with antibodies against Dek and K5, then stained with DAPI to detect DNA. As expected, Dek expression was higher in the *Bi-L-Dek_K5-tTA* derived keratinocytes compared to those treated with dox, or compared to keratinocytes from single transgenic control mice (**Fig 2F and 2G**). Exogenous Dek localized to the nucleus as expected, and co-localized with endogenous Dek and DAPI (**Fig 2F**). Altogether, *Bi-L-Dek_K5-tTA* mice overexpressed Dek in the squamous epithelium of the skin, tongue, and esophagus, and Dek expression was repressed by doxycycline.

**Fig 3:**
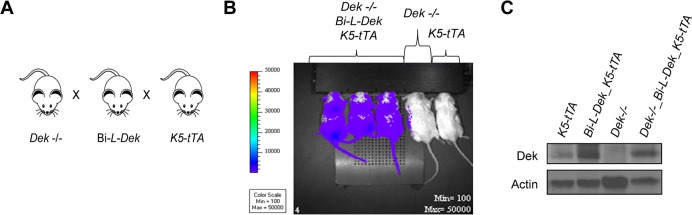
*Bi-L-Dek* transgene expression is detected in the context of Dek knockout mice. (**A**) *Bi-L-Dek_K5-tTA* mice were bred to Dek knockout (*Dek-/-)* mice to quantify Dek expression in the absence of endogenous Dek protein. (**B**) IVIS image of *Dek-/-* _*Bi-L-Dek_K5-tTA* mice with luciferase expression compared to *Dek-/-* and single transgenic *K5-tTA* mice after luciferin injection. (**C**) Western blot analysis detects Dek protein expression in murine flank skin from *Dek-/-* _*Bi-L-Dek_K5-tTA* mice.

To assess the extent of Dek overexpression in the *Bi-L-Dek_K5-tTA* mice, we quantified transgene expression in the context of Dek knockout mice. *Bi-L-Dek* and *K5-tTA* transgenic mice were interbred with *Dek-/-* mice to generate *Dek-/-*_ *Bi-L-Dek_K5-tTA* offspring (**Fig 3A**). IVIS confirmed luciferase expression (**Fig 3B**), and RT-qPCR and western blot analysis confirmed Dek mRNA and protein expression, respectively, in the epidermis (**Fig 3C and S2 Fig**). Dek mRNA levels were induced by approximately four fold in the *Bi-L-Dek_K5-tTA* compared to control mice (**S2 Fig**) and Dek protein levels were induced by approximately 2–3 fold in the *Bi-L-Dek_K5-tTA* over control mice. These levels are similar to the levels of DEK expression that can be routinely achieved in normal epithelial cells transduced with retroviral or lentiviral DEK expression vectors and are within the range of DEK levels observed in cancer cells [16, 42, 56, 77–81].

### Brain-specific and global *Bi-L-Dek* transgene expression is achievable via *Dlx5/6-tTA* and *Rosa-tTA* drivers

To explore the broader utility of this model system, conditional *Bi-L-Dek* mice were bred to *Dlx5/6-tTA* mice to target Dek expression to neurons originating from the ventral forebrain (**S3 Fig**). As expected, robust Dek protein overexpression was detectable in cortical interneurons and striatal projection neurons from the ventral forebrain, as previously demonstrated for other Dlx5/6-driven transgenes [82]. Additionally, we crossed the *Bi-L-Dek* mice with *Rosa-tTA* mice to produce global Dek overexpressing mice. IVIS demonstrated luciferase expression from the *Bi-L-Dek* transgene throughout the body, which was repressed with dox chow (**S4 Fig**). No overt phenotypes were observed in the mice similar to results in previously published *Tet-O-Dek*_ *Rosa26-M2rtTA* mice. In all, these results further validate *Bi-L-Dek*-mediated transgene expression in murine epithelia, and demonstrate broad utility of this genetic mouse model for studies of Dek overexpression in other organ systems.

### Dek overexpression promotes esophageal squamous cell carcinoma

**Fig 4:**
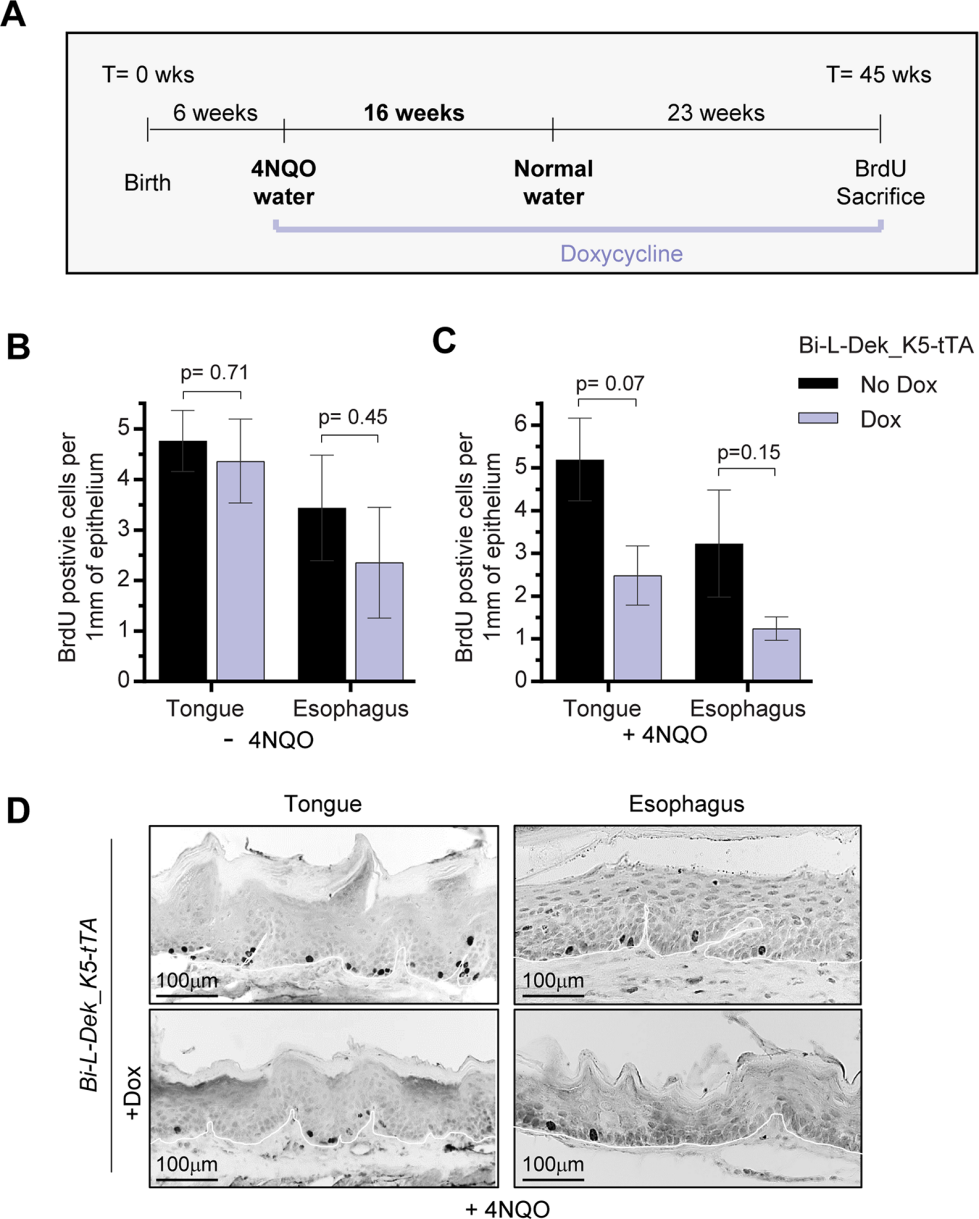
Dek overexpression in 4NQO-treated mice leads to trends in increased cellular proliferation. (**A**) The water soluble carcinogen 4-nitroquinoline 1-oxide (4NQO) confers susceptibility to head and neck squamous cell carcinoma (HNSCC). At six weeks of age, mice were given 4NQO drinking water for 16 weeks in the presence or absence of dox chow. After 16 weeks, the mice were returned to normal water and were monitored until death or sacrifice at 45 weeks of age, or earlier if moribund. Prior to sacrifice, mice were injected with BrdU to measure proliferative differences in tissues. Abbreviations: time (T), weeks (wks). (**B-C**) Quantification of BrdU positive cells per millimeter of squamous epithelium in (**B**) normal and (**C**) 4NQO treated tongue and esophagus of *Bi-L-Dek_K5-tTA* mice in the absence or presence of dox. Three to six mice were examined for each tissue in the no dox/dox treatment groups and a student’s t-test was used to determine statistical significance. A minimum of 100mm of epithelium was quantified per mouse and epithelial distance was measured using ImageJ [89]. (**D**) Representative IHC images of increased BrdU incorporation in the tongue and esophagus of *Bi-L-Dek_K5-tTA* mice treated with 4NQO compared to those on dox.

**Fig 5:**
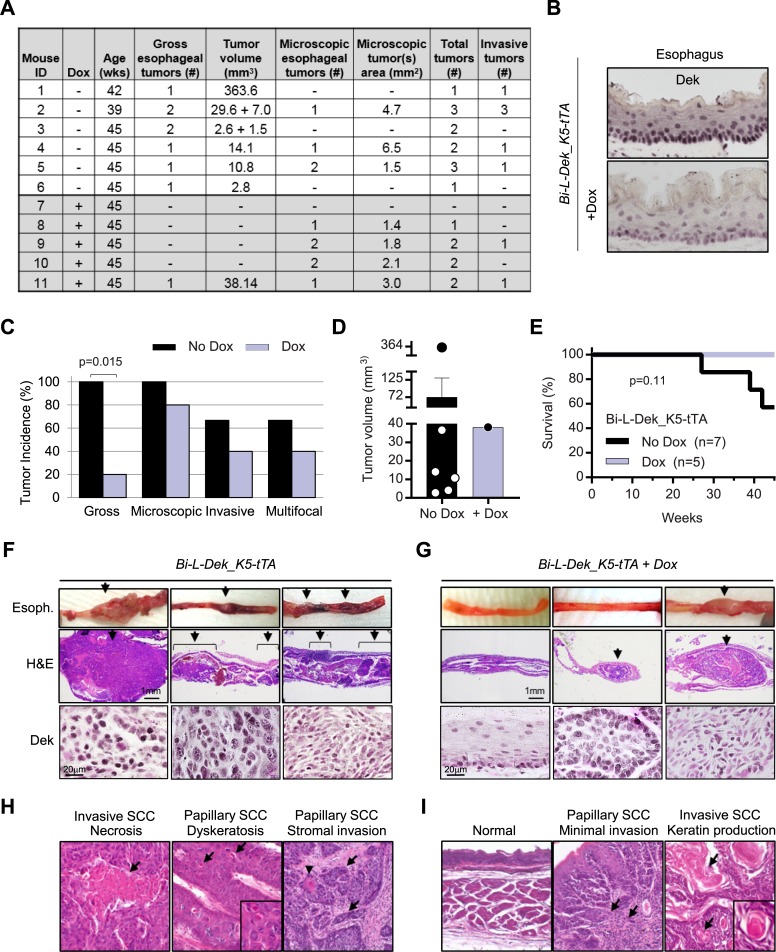
Dek overexpression increases the incidence of gross esophageal tumors. (**A**) Details on mice and pathologies including esophageal tumor volumes in the 4NQO-treated mice. (**B**) Representative IHC images for Dek protein overexpression in the esophagus of *Bi-L-Dek_K5-tTA* mice treated with 4NQO compared to mice on dox (Dek antibody: Cusabio, Balitmore, MD, USA; magnification: 40x). (**C**) Percent incidence of gross, microscopic, invasive, and multifocal tumors within the two groups of mice. Statistics is indicated when significantly different between the no dox/dox treated groups as determined by a Fisher Exact test. (**D**) Gross tumor volumes within the two groups. Each dot represents total gross tumor volume per mouse (no statistics due to an n = 1 for the no dox group). (**E**) Survival of the *Bi-L-Dek_K5-tTA* Dek overexpressing mice +/- dox treatment. Tissue from a seventh *Bi-L-Dek_K5-tTA* mouse that died at 27 weeks could not be evaluated for tumors at necropsy (not included in Fig 5A). (**F-G**) Images of esophagi at the time of dissection (top), and the corresponding H&E stained histologic sections of esophagus (middle), and Dek staining by IHC in the corresponding tumor (bottom) from *Bi-L-Dek_K5-tTA* mice in the absence (**F**) or presence (**G**) of dox (H&E magnification: 2x; Dek IHC magnification: 120x; Dek antibody: Cusabio, Baltimore, MD, USA). (**H-I**) Images of H&E stained esophageal sections illustrate morphological features of tumors in (**H**) Dek overexpressing *Bi-L-Dek_K5-tTA* mice and (**I**) normal esophagus and tumors in dox treated *Bi-L-Dek_K5-tTA* mice. Extensive necrosis in a poorly differentiated invasive squamous cell carcinoma (H, left panel arrows), and dyskeratotic cells (H, middle panel arrows) along with cellular dysplasia and intercellular bridges (H, middle panel inset), and extensive stromal invasion (H, right panel arrows) with focal squamous differentiation (H, right panel arrowhead) in papillary squamous cell carcinoma in Dek overexpressing mice are shown. Esophageal images from dox treated *Bi-L-Dek_K5-tTA* mice illustrate the normal esophagus from mouse lacking tumors (I, left panel), a microscopic papillary squamous cell carcinoma with minimal superficial stroma invasion (I, middle panel, arrows), and the single grossly apparent tumor characterized as a well differentiated invasive squamous cell carcinoma with abundant keratin production (I, right panel, arrows and inset). (Original magnifications: 40x, inserts 100x).

Based on data in the cancer genome atlas (TCGA), DEK is more highly expressed in ESCC compared to normal tissue and in ESCC compared to esophageal adenoma (S5 Fig). However, the contribution of Dek overexpression to ESCC development is unknown. To determine if DEK contributes to ESCC development or progression, we utilized the *Bi-L-Dek_K5-tTA* mice with Dek overexpression targeted to basal keratinocytes that form the epithelium. *Bi-L-Dek_K5-tTA* mice overexpressed Dek in stratified squamous epithelium of the tongue and esophagus, and *Bi-L-Dek_K5-tTA* mice on dox expressed only endogenous levels of Dek. Exposure of mice to drinking water containing the soluble quinoline derivative 4NQO promotes the development of oral and/or esophageal cancer. Therefore, we exposed two groups of mice +/- Dox to 4NQO in order to determine whether Dek overexpression in basal keratinocytes is sufficient to promote SCC and if early onset of Dek overexpression increases oral and/or esophageal tumor incidence or tumor burden. The experimental design is illustrated in **Fig 4A**. At six weeks of age, *Bi-L-Dek_K5-tTA* mice in the absence of dox (n = 7) or in the presence of dox (n = 5) were exposed to 10ug/mL of 4NQO in their drinking water to promote SCC susceptibility. After 16 weeks, mice were given normal water, sacrificed at 45 weeks of age or when moribund, and analyzed after experimentally induced death or sacrifice. One hour prior to sacrifice, mice were injected with the thymidine analog Bromodeoxyuridine (BrdU) to quantify proliferation. In the absence of 4NQO treatment, Dek overexpression did not significantly increase cellular proliferation in the tongue or the esophagus when compared to control mice on dox (**Fig 4B**). However, following 4NQO treatment there was a trend toward increased proliferation in the epithelia of Dek overexpressing tongue (p = 0.07) and esophagus (p = 0.15) (**Fig 4C and 4D**). These results are in line with recently published data wherein global Dek overexpressing mice did not exhibit hyperplasia or other phenotypes under normal conditions, but displayed tongue hyperplasia after 4NQO treatment [72]. *Bi-L-Dek_K5-tTA* mice exposed to 4NQO or not were then analyzed for the presence of tumors in the tongue and esophagus. Detailed results are shown for each mouse in **Fig 5A**. After 4NQO treatment, *Bi-L-Dek_K5-tTA* mice continued to express higher levels of Dek protein in esophageal epithelium compared to the control group on dox (**Fig 5B**). In addition, Dek overexpressing mice had a significantly higher incidence of gross esophageal tumors (**Fig 5C**). Specifically, all of the *Bi-L-Dek_K5-tTA* mice developed at least one visible esophageal tumor (100%), in contrast to only one of five *Bi-L-Dek_K5-tTA* mice on dox (20%). Furthermore, one Dek overexpressing mouse harbored an excessively large tumor, while two others harbored two separate grossly apparent tumors (**Fig 5A, 5C and 5D**). From published studies, the 4NQO protocol utilized was expected to result in a 10% incidence of gross tumors in *Dek* wild type mice [75, 83–85]. This compares roughly to the 20% cancer incidence in mice exposed to dox. Overall, the number of invasive tumors and mice with multifocal tumors was not significantly different between the two groups (**Fig 5C**); however, survival of Dek overexpressing mice was less than 60% while all dox-treated mice survived until sacrifice at week 45 (p = 0.11; **Fig 5E**). Taken together, these data provide evidence that Dek overexpression promotes esophageal squamous cell carcinoma growth.

Esophagi and tongues from all mice were microscopically examined to define tumor phenotypes and quantify microscopic lesions. Histological analysis confirmed that gross tumors in Dek overexpressing mice were squamous cell carcinomas with stromal invasion confirmed histologically in 67% of the mice (**Fig 5A, 5C and 5H**). Additional multifocal microscopic squamous cell lesions were detected in 50% of Dek overexpressing mice with all mice developing 1–3 squamous cell lesions including at least one grossly apparent tumor. In contrast, microscopic lesions predominated in dox treated mice, with one mouse harboring no lesions and the other mice harboring 1–2 squamous cell lesions including a single grossly apparent tumor (**Fig 5A, 5G and 5I**). The single tumor apparent at necropsy in this group was a well differentiated squamous cell carcinoma with abundant keratin production which differed from the moderate to poorly differentiated squamous cell carcinomas that predominated in Dek overexpressing mice (**Fig 5I and 5H**). Microscopic tumors in dox treated mice consisted primarily of papillary squamous cell lesions with a single focus of very superficial invasion in one lesion. This differed from the more extensive invasion and necrosis in tumors that arose in Dek overexpressing mice (**Fig 5I and 5H**). Dek levels appeared to be high in all tumors regardless of whether these originated in the Dek overexpressing group or the dox control group, thus suggesting strong selection for the upregulation of endogenous Dek during tumorigenesis (**Fig 5F and 5G**, bottom row). With regards to the one tumor that arose in the dox control group, endogenous upregulation or leaky transgenic expression of Dek could be responsible. No tongue tumors were identified in either group of mice. Taken together, we demonstrate for the first time that Dek overexpression promotes the growth of esophageal SCC *in vivo*.

## Discussion

A number of studies have linked DEK overexpression in various malignancies to cellular growth, motility/invasion and chemoresistance [16, 36, 37, 43, 58, 71, 77]. Relevant mechanisms have not been fully elucidated in each case. However, DEK loss has been shown to attenuate proliferation and survival, while inducing senescence or apoptosis, depending upon the cell type and model system studied. Required signaling pathways included those controlled by p53 and ΔNp63 to inhibit apoptosis and promote proliferation, respectively [17, 39, 58], Wnt/beta-catenin to drive invasion and cellular proliferation [32], VEGF to foster angiogenesis [7], Rho/ROCK/MLC to support migration [86], and NFkB to regulate cellular survival and growth [6, 8, 59]. One caveat regarding cancer-related interpretation of these results is that many of the experiments are based upon DEK loss of function, and thus only address the requirement for DEK in tumor cell growth and not the contribution of DEK overexpression to tumor growth. For instance, Dek knockout mice are viable and resistant to chemically induced papillomas and HPV E7 driven HNSCC. *In vitro*, DEK overexpression in primary keratinocytes extends life span, stimulates transforming activities of classical oncogenes, and de-regulates cellular metabolism [16, 17, 78]. These data are in line with, but do not prove, oncogenic activities that promote cancer development at the organismal level.

Here we demonstrate that Dek overexpression targeted to the epithelium stimulates proliferation specifically in the presence of 4NQO in the tongue and also in the esophagus. Furthermore, concurrent Dek overexpression and 4NQO exposure increased the incidence of gross esophageal tumors demonstrating for the first time that Dek overexpression contributes to ESCC tumor growth *in vivo*. The observed increase in hyperplasia in the tongue is similar to that seen with sequential 4NQO exposure followed by ubiquitous Dek overexpression reported by Nakashima et. al.(72). Interestingly, and in contrast to our data, Nakashima et. al. reported that Dek overexpression decreased the volume of resulting tongue tumors [72]. Key differences in the experimental designs between the two models likely account for the observed differences in tumor location and size in. Specifically, in the current study: 1) Dek overexpression was targeted to the basal epithelium as opposed to ubiquitous Dek overexpression including immune and stromal cells that modulate cancer cell growth, 2) 4NQO and Dek overexpression were concurrently administered rather than sequential exposure to 4NQO followed by Dek overexpression, 3) 4NQO exposure duration and dosage was 16 weeks at 10 μg/ml compared to 28 weeks at 20 μg/ml, 4) Dek overexpression duration was 52 weeks compared to four weeks, 5) exogenous Dek was unmodified and localized to the nucleusas compared to FLAG-tagged exogenous Dek protein localized predominantly to the cytoplasm, and 6) FVB/N mice were used compared to C57BL/6 mice. Interestingly, C57BL/6 and FVB/N harbor variations in immune phenotype, raising the intriguing possibility that immune surveillance and/or evasion account at least in part for the differing tumor phenotypes in mice with ubiquitous versus epithelial cell targeted Dek overexpression. Preliminary studies in the esophageal tumors in the current model did not reveal a significant CD3 positive T cell infiltrate by immunohistochemistry. Additional studies are needed to definitely determine the role of inflammatory cells in Dek dependent tumorigenesis, however, the lack of a prominent T-cell infiltrate suggests that differences in tumor growth in the two models cannot be simply explained by tumor infiltrating T cells acquiring an exhausted T-cell phenotype. The availability of these distinct complementary mouse models now provide a valuable system to identify cell specific functions that drive Dek induced carcinogenesis.

Distinct effects of global versus tissue-specific Dek expression might reflect interesting cell-type specific functions of Dek in the tumor microenvironment including immune cells, or systemic effects on epidermal proliferation and tumor growth. The complexity of DEK functions in vivo is exemplified in studies of non-vertebrate organisms For instance, in Arabidopsis, DEK3 overexpression decreases germination efficiency under high salinity conditions, and conversely, plants deficient in DEK3 germinated significantly better compared to wild-type plants suggesting DEK3 levels are crucial for stress tolerance [87]. The overexpression of human DEK in the Drosophila eye caused a rough-eye phenotype due to caspase-9 and 3-mediated apoptosis suggesting that DEK overexpression caused (rather than diminished) apoptosis [88]. These non-vertebrate eukaryote model systems highlight the need for balanced DEK expression and its versatile functions *in vivo*.

In the *Bi-L-Dek_K5-tTA* mouse model, Dek overexpression at the message and protein level was approximately 2–4 fold over that of endogenous Dek. This relatively modest level is in agreement with other published studies suggesting DEK expression levels are tightly regulated [1, 16, 58, 77–79]. Achieving strong overexpression of DEK *in vitro* in our hands has been notoriously difficult, potentially due to toxicity and cell death, e. g. in the above Drosophila study [88]. Importantly, a modest level of DEK overexpression in epithelial cells has been linked to oncogenic phenotypes *in vitro*. These DEK dependent oncogenic activities include enhanced cancer stem cell growth, colony formation, cellular invasion, mitotic abnormalities, and metabolic de-regulation, providing evidence that subtle increases in DEK protein expression are sufficient to elicit significant cellular consequences [16, 77–79]. In human ESCC, HNSCC, breast, bladder, colorectal, hepatocellular, and non-small cell lung carcinoma, DEK protein levels were increased in tumor versus adjacent normal tissue, and the extent of overexpression was variable. Per cell DEK protein detection in various tumor types can range from intense to weak staining by IHC, and overexpression by western blot analysis can range from 2–30 fold [23, 25, 26, 31, 34, 39, 42, 77, 80, 81]. Overall, this patient data suggests that high levels of DEK can be tolerated by some human tumor cells, and that even modest DEK expression is associated with cancer growth and/or maintenance.

In conclusion, *Bi-L-Dek_K5-tTA* mice subjected to 4NQO harbor trends toward increased cellular proliferation in the tongue and esophagus (**Fig 4B–4D**) and a significantly increased incidence of gross esophageal tumors (**Fig 5C**). Tongue tumors were not detected in these same mice. Importantly, control *Bi-L-Dek_K5-tTA* mice on dox nonetheless developed microscopic ESCC tumors, thus suggesting that Dek overexpression does not stimulate tumor initiation, but promotes tumor growth in the esophagus. This is in alignment with previously published Dek loss of function data from HNSCC-prone K14E7 transgenic mice wherein keratinocyte proliferation and tumor growth, but not the presence of microtumors, were diminished in the absence of Dek [39]. While an abundance of data has suggested that DEK promotes tumor growth in the presence of oncogenic stimuli, the above experiments do not unequivocally rule out a role for Dek in tumor initiation. Overall larger tumors in the Dek overexpressing mice may be due to increased growth of tumors once initiated, or due to premature initiation and thus extended time for growth. In either case, Dek overexpression significantly increased the incidence of gross tumors and over 40% of *Bi-L-Dek_K5-tTA* mice died prior to the 45 week end point, while all mice in the dox treated control group survived. A plethora of Dek knockdown experiments have shown the importance of DEK expression for cancer cell growth and survival [10, 16, 39, 58, 77]. These data, in conjunction with evidence that transformed keratinocytes are more sensitive to DEK loss when compared to their normal or differentiated counterparts [16], make DEK an attractive therapeutic target. Furthermore, Dek knockout mice are healthy and fertile, suggesting potential feasibility and relative safety for the targeting of DEK in cancer. However, no DEK inhibitors exist commercially nor have been published. Thus, the inducible targeting of Dek in *Bi-L-Dek* mice harboring ESCC tumors should now be an attractive model to interrogate the requirement of continued Dek expression for cancer maintenance and progression. Taken together, we have generated and validated a new mouse model of esophageal transformation using an inducible *Bi-L-Dek* transgene which is now available for broader studies of Dek in health and disease of the intact organism.

## Materials and methods

### Generation of *Bi-L-Dek* transgenic mice

Murine Dek (mDek) DNA sequences were excised from the previously published R780 retroviral vector, using the restriction enzymes Sal I and Not I [16, 71], and cloned into the pBi-L plasmid (Clontech, Mountainview, CA Catalog No. 631005; GenBank Accession No.: U89934.) cleaved with the same restriction enzymes. The resulting *pBi-L-Dek* construct harbors the bi-directional Pbi-1 promoter which is responsive to the tTA regulatory protein in this Tet-Off system. The Tet-responsive element (TRE) consists of seven copies of the 42-bp tet operator sequence (tetO), and is located between two minimal CMV promoters that lack the CMV enhancer. Gene expression is silent in the absence of the tTA bound to tetO sequences and is silenced with the addition of doxycycline. The p*Bi-L-Dek* transgene sequences were liberated using the restriction enzymes AatII and AselI. A 5247bp (*Bi-L-Dek*) DNA sequence was purified and microinjected into the pronucleus of a fertilized egg and inserted into a pseudo-pregnant mouse to produce *Bi-L-Dek* founders. Transgene transmission was validated, and pups from the F1 generation were mated with *K5-tTA* mice. Resulting F2 *Bi-L-Dek_K5-tTA* mice were further characterized. Four *Bi-L-Dek* founders were generated. One founder line never produced offspring. Another founder died before producing a pup that harbored the transgene. Of the two remaining lines, both overexpressed Dek but founder #317 was a better breeder. The murine Dek sequence that was cloned into the pBi-L-Tet vector is:

5’-ATGTCGGCGGCGGCGGCCCCCGCTGCGGAGGGAGAGGACGCCCCCGTGCCGCCC TCATCCGAGAAGGAACCCGAGATGCCGGGTCCCAGGGAAGAGAGTGAGGAGGAGGAGGAGGATGACGAAGACGATGATGAAGAGGACGAGGAGGAAGAAAAAGAAAAGAGTCTTATCGTGGAAGGCAAGAGAGAGAAGAAGAAAGTAGAGAGACTGACGATGCAAGTGTCTTCCTTACAGAGAGAGCCATTTACAGTGACACAAGGGAAGGGTCAGAAACTTTGTGAAATTGAAAGGATACATTTCTTTCTGAGTAAGAAAAAACCAGATGAACTTAGAAATCTACACAAACTGCTTTACAACAGGCCGGGCACAGTGTCCTCGTTGAAGAAGAACGTGGGTCAGTTCAGTGGCTTTCCATTCGAAAAAGGCAGTACCCAGTATAAAAAGAAGGAAGAAATGTTGAAAAAGTTTCGAAATGCCATGTTAAAGAGCATCTGTGAGGTTCTTGATTTAGAGAGGTCAGGCGTGAACAGCGAACTCGTGAAGAGGATCTTGAACTTCTTAATGCATCCAAAGCCTTCTGGCAAACCATTACCAAAGTCCAAAAAATCTTCCAGCAAAGGTAGTAAAAAGGAACGGAACAGTTCTGGAACAACAAGGAAGTCAAAGCAAACTAAATGCCCTGAAATTCTGTCAGATGAGTCTAGTAGTGATGAAGATGAGAAGAAAAATAAGGAAGAGTCTTCGGAAGATGAAGAGAAAGAAAGTGAAGAGGAGCAACCACCAAAAAAGACATCTAAAAAAGAAAAAGCAAAACAGAAAGCTACTGCTAAAAGTAAAAAATCTGTGAAGAGTGCTAATGTTAAGAAGGCAGACAGCAGTACCACCAAGAAGAATCAAAAAAGTTCCAAAAAAGAGTCTGAATCCGAAGACAGTTCTGATGATGAACCCTTAATTAAAAAATTGAAAAAGCCACCTACAGATGAAGAGCTAAAGGAAACAGTGAAGAAATTACTGGCTGATGCTAACTTGGAAGAAGTCACAATGAAGCAGATTTGCAAAGAGGTATATGAAAATTATCCTGCTTATGATTTGACTGAGAGGAAAGATTTCATTAAAACAACTGTAAAAGAGCTAATTTCTTGA-3’

### Genetic mouse models

*K5-tTA* mice were obtained internally at CCHMC and have previously been published [90]. Dek knockout mice (*Dek-/-*) have previously been published [16]. *Dlx5/6-tTA* mice were obtained internally at CCHMC and were generated in Dr. Kenneth Campbell’s lab by Lisa Ehrman. *Dlx5/6-tTA* mice have been analyzed for tTA expression, and will be fully described and characterized in a separate publication. *Dlx5/6* tTA expression is similar to Cre expression in the reported *Dlx5/6-*Cre-IRES-EGFP *(CIE)* transgenic mouse model [82]. *E2A-Cre* mice were obtained from Jackson Laboratory and are strain number 003724. The Cre transgene is under the control of the adenovirus EIIa promoter, which targets expression of Cre recombinase to the early mouse embryo. This model is useful for deletions, in the germ line, of *loxP*-flanked genes. *E2A-Cre* mice were bred to *Rosa-LNL-tTA* transgenic mice. These mice were also obtained from Jackson laboratories and are strain number 008600. Rosa-LNL-tTA mice contain a loxP-flanked nonsense sequence inhibiting expression of tTA that is removed once exposed to E2A controlled Cre.

### Genotyping

Ear clips were digested with 25mM NaOH in 0.2mM EDTA at a pH of 12 and incubated at 95°C for 20 minutes. The reaction was neutralized with 40mM Tris-HCl. For PCR analysis, one ul of the digest with DNA was added to JumpStart Taq Ready Mix from Invitrogen (Carlsbad, CA, product # P2893) using the manufacturer’s specifications. Transgenes were detected with the following primers:

*Bi-L-Dek*:

Forward: GAAATGTCCGTTCGGTTGGCAGAAGC;

Reverse: CCAAAACCGTGATGGAATGGAACAACA.

*K5-tTA*:

Forward: GCTGCTTAATGAGGTCGG

Reverse: CTCTGCACCTTGGTGATC.

*Bi-L-Dek* primers that do not detect endogenous Dek (exogenous Dek cDNA primers)

Forward: CAGTGACACAAGGGAAGGGTCAGA

Reverse: AGCCACTGAACTGACCCACGT.

### *Bi-L-Dek* copy number determination by qPCR

Genomic DNA was isolated from the tails of mice from successive generations of offspring from founder 317. A minimum of two mice were used per generation and analyzed as replicates. The DNA concentration was adjusted to 20ng/ul in each case, and 60ng of DNA was used for qPCR per sample and performed in duplicate. Primers were used to quantify the beta actin gene and a region in exon 6 of the Dek gene. This region is present in Dek+/+ mice but absent in *Dek-/-* mice thus allowing for a negative control. The following sequences were used:

Beta actin forward: GATATCGCTGCGCTGGTCGTC

Beta actin reverse: ACCATCACACCCTGGTGCCTAG

Dek Exon 6 forward: AGGTCAGGCGTGAACAGCGA

Dek Exon 6 reverse: TGCCAGAAGGCTTTGGATGCATTA

The critical threshold (CT) values for Dek exon 6 primers were normalized to actin, and quantified relative to Dek wild type mice using the delta delta CT method. Values were multiplied by two to account for the two endogenous Dek alleles in WT mice and the number of *Bi-L-Dek* transgene insertions was determined. Error bars represent multiple mice from the same generation.

### *In vivo* Imaging Systems (IVIS)

Mice were injected with 15ng of luciferin per gram in body weight, and allowed to metabolize the luciferin for five minutes prior to sedation with isoflurane. Mice were imaged in the Perkin Elmer IVIS Spectrum CT, Waltham, Massaschusetts, USA. For *ex vivo* IVIS, mice were allowed to metabolize luciferin for eight minutes following luciferin injection, and then sacrificed with CO2. The mice were then dissected and tissues placed in PBS containing 300ug/mL of luciferin, kept on ice, and protected from light before immediate analysis by IVIS.

### Mouse keratinocyte culture

For validation of p*Bi-L-Dek* expression, the plasmid was transfected into previously isolated and cultured *K5-tTA* expressing murine keratinocytes [91]. Cells were collected for Dek protein expression by western blot analysis. *K5-tTA* keratinocytes were grown in E-media supplemented with 0.05 mM Ca2 and 15% serum as previously published [92].

Keratinocytes were isolated from *Bi-L-Dek_K5-tTA* mice and single transgenic littermate controls using a previously published protocol with modifications [93]. Briefly, pups were euthanized within 48 hours of birth, rinsed in 70% ethanol, and placed in PBS. Flank skin was removed, and placed dermis side down in 1 mL of dispase (Dispase Gibco/Invitrogen, Calsbad, CA, USA, product# 17105–041) and 1 mL of DMEM (1:1 mixture) in a 35mm plate, and incubated overnight at 4° Celsius. The epidermis was removed and placed in 1 mL of accutase (Sigma, St. Louis, MO, USA, product # A6964) for 20 minutes with agitation to release the keratinocytes. Cells were collected and centrifuged, then plated on irradiated MEFs and overlaid with CnT07 media (CellnTec, Bern, Switzerland). Cells were used for experiments in passage 0 or 1.

### Immunofluorescence microscopy

Keratinocytes were plated onto 100 mg/ml poly-D-lysine coated coverslips, and fixed with 2% paraformaldehyde for 30 minutes. Coverslips were incubated in 0.1% Triton X-100 for three minutes, blocked with 5% normal goat serum, and incubated with primary antibody for one hour at 37°C. Antibody dilutions were as follows: DEK-antibody (Cusabio, Baltimore, MD, USA) 1:300 dilution; keratin 5 antibody (Acris, San Diego, CA, USA) 1:500; and sealed with a coverslip using Vectashield with DAPI (Vector Laboratories, Burlingame, CA). ImageJ (National Institutes of Health, Bethesda, Maryland, USA) [89] was used to quantify Dek staining. Dek immunofluorescences (IF) images were converted to 8-bit images, followed by the identification of the location of cells with the nucleus counter ImageJ plugin. Each cell was visually validated and added to the regions of interest (ROI). The mean grayscale intensity was measured in these ROIs. Quantification was from successive images to encompass the entire coverslip of keratinocytes isolated from each genotype with or without dox treatment.

### Western blot analyses

Tissues were lysed using mortar and pestle, resuspended in RIPA buffer (1% Triton, 1% deoxycholate, 0.1% SDS, 0.16M NaCl, 10 mmol/L Tris pH 7.4, and 5 mmol/L EDTA), supplemented with a protease inhibitor cocktail (Pharmingen, San Diego, CA, USA), and analyzed as described previously (48). Primary antibodies used for DEK were as follows: DEK (1:1000; BD Biosciences, San Diego, CA, USA), pan-actin (1:20,000; a gift from James Lessard). Membranes were exposed to enhanced chemiluminescence reagents (Perkin Elmer, Boston, MA, USA) and imaged using the BioRad Chemidoc (Hercules, CA, USA).

### 4NQO induction of HNSCC development *in vivo*

All mice were maintained in a hemizygous state for the *Bi-L-Dek* and *K5-tTA* transgenes. All *Bi-L-Dek* mice were F3 and F4 generations from founder 317. *Bi-L-Dek* mice were bred to *K5-tTA* mice and bi-transgenic offspring were given 4NQO water for 16 weeks at a dose of 10mg/ml starting at six weeks of age. Mice on doxycycline were continuously fed dox chow from the start of 4NQO treatment until sacrifice. After 16 weeks on 4NQO, mice were returned to normal water until sacrifice at week 45 or when determined excessively morbid by veterinary services thus warranting sacrifice. At the time of sacrifice, tumors were resected and counted, localization was noted, and tumors were measured by calipers. Tumor volume was measured by (length x width x depth). All statistical analyses were performed in GraphPad Prism. The survival curve was analyzed using the log-rank (Mantel-Cox) test. Tumor incidence was determined significant/non-significant using the Chi Square (and Fisher’s exact) test.

### Histological analyses and immunohistochemistry

Mouse tumors and tissues were fixed in 4% paraformaldehyde, embedded in paraffin, sectioned at 5 μm thickness, and fixed onto slides. Routine H&E stained sections were analyzed for histopathology.^13^ The area of microscopic tumors was determined by multiplying the widest part of the tumor by the longest part that was observed in the sections. Paraffin sections were deparaffinized in xylene and rehydrated for antigen retrieval in sodium citrate. Sections were then treated with the Mouse on Mouse peroxidase immunostaining kit (Vector Labs, Burlingame, CA, USA). Sections were stained with diaminobenzidine (DAB) and counterstained with Nuclear Fast Red (Poly Scientific, Bay Shore, NY, USA) and mounted with Permount (Fisher Scientific, Pittsburgh, PA, USA). Images were captured at the indicated magnifications and antibodies used are noted in each case. Antibody dilutions were used as follows: BrdU (1:100, Invitrogen, Calsbad, CA, USA), and DEK (1:200, BD Biosciences, San Jose, CA, USA; or 1:300, Proteintech Group, Chicago, IL, USA; or 1:50, Cusabio, Baltimore, MD, USA).

### BrdU quantification

10x or 20x magnified images of BrdU stained tongue or esophagus were analyzed for BrdU positive cells using ImageJ (National Institutes of Health, Bethesda, Maryland, USA). In ImageJ, the bottom portion of the basal cell layer of the stratified squamous epithelium was traced using the freehand tool and measured in the indicated tissue. The distance was converted into millimeters using scale bars based on magnification to determine BrdU positive cells per millimeter of epithelium. Statistical analysis was performed using GraphPad Prism with t-tests and the two-stage linear step-up procedure of Benjamini, Krieger and Yekutieli.

### Luciferase experiments

Luciferase assays were performed using the Dual-Luciferase Reporter Assay System from Promega and following manufacturer specifications.

### Ethics statement

All animal work was conducted according to Cincinnati Children's Hospital Medical Center Institutional Animal Care and Use Committee guidelines under protocol number #2017–0004.

To ameliorate animal suffering mice were euthanized with carbon dioxide when moribund as determined by veterinary services.

## Supporting information

S1 Fig Generation of tetracycline responsive *Dek* transgenic mice. (**A**) Murine Dek cDNA was cloned into the pBi-L-tet plasmid (Clontech, Mountain View, CA, USA) as described in the Materials and Methods. Therein, Dek and luciferase expression are under control of a tetracycline response element (TRE). Restriction enzyme sites for AatII and AseI were used to excise the transgenic construct. (**B**) Western blot analysis for Dek expression in mouse keratinocytes isolated from a *K5-tTA* mouse (84) and transfected with the p*Bi-L-Dek* plasmid. Dek protein expression was repressed with 1ug/ml of dox in the media. (**C**) Luciferase assay with keratinocytes from B, treated with 0, 0.33 or 1.5 ug/ml of dox show dose dependent luciferase repression. (**D**) Agarose gel electrophoresis of the p*Bi-L-Dek* plasmid linearized with restriction enzymes AseI and AatII and the resulting 5247bp *Bi-L-Dek* band. (**E**) The 5247bp *Bi-L-Dek* band was isolated from the gel, purified, and used for micronuclear injection into the pronucleus of FVB/N fertilized eggs to generate *Bi-L-Dek* founders. (**F**) Four founders were generated as confirmed by genotyping with primers that detect luciferase and exogenous Dek cDNA sequences. Founder #317 was used for subsequent experiments.
(EPS)

S2 Fig Detection of Dek mRNA expression from the *Bi-L-Dek* transgene. RT-qPCR for quantification of relative Dek transcript levels in *Dek-/-*, *Dek+/+*, and *Dek-/-*_*Bi-L-Dek_K5-tTA* (black bar) flank tissue (n = 1 mouse per genotype). Primers detect a cDNA region present in endogenous and *Bi-L-Dek* mice that is absent in the *Dek-/-* mice (Dek exon 6 primer set in methods and materials).
(EPS)

S3 Fig *Bi-L-Dek* transgene expression targeted to the brain. (**A**) *Dlx5/6-tTA* mice were crossed with the *Bi-L-Dek* mice. The *Dlx5/6* enhancer sequences drive tTA expression in neurons that originate in the ventral forebrain (**B**) Genotyping confirmed transgene transmission to offspring near expected ratios. Lanes 1–6 represent offspring of the cross from A. Lane 1 represents a bi-transgenic mouse that was used for Dek expression studies in C. (**C**) IHC for Dek in the brains of pups at embryonic day 18 shows Dek overexpression in the *Dlx5/6-tTA_Bi-L-Dek* mouse cortex and striatum. Insets are magnified images of the cortex and striatum where Dek is overexpressed (Dek antibody: Proteintech Group, Chicago, IL, USA).
(EPS)

S4 Fig Doxycycline responsive, whole body *Bi-L-Dek* transgene expression. (**A**) To generate mice with whole body Dek overexpression, we bred *Bi-L-Dek* mice to *Rosa-tTA* mice. To generate *Rosa-tTA* mice, *E2A-Cre* mice were bred to Rosa-LNL-tTA mice. *E2A-Cre* mice carry a Cre transgene under the control of the adenovirus EIIa promoter that targets expression of Cre recombinase to the early mouse embryo. *Rosa-LNL-tTA* mice harbor a *tTA* transgene in the Rosa 26 locus that is preceded by a stop codon flanked by *loxP*- sequences (LNL) which inhibits tTA translation. The *E2A-Cre* mice were used for germ line deletion of the *LNL* resulting in *Rosa-tTA* mice with global expression of tTA. (**B**) Genotyping of the offspring shows excision of the *LNL* and transmission of the *Rosa-tTA* and *Bi-L-Dek* transgenes. Mouse 1 and 3 have both *Bi-L-Dek* and *Rosa-tTA* transgenes as indicated by red arrows. (**C**) *Bi-L-Dek_Rosa-tTA* mice 1 and 3 along with a single transgenic littermate were subjected to IVIS at six weeks of age for luciferase detection. (**D**) Mice were placed on dox for two weeks and subjected to IVIS again to show repression of luciferase expression from the *Bi-L-Dek* transgene in *Bi-L-Dek_Rosa-tTA* bi-transgenic mice.
(EPS)

S5 Fig DEK is overexpressed in esophageal cancer. (A) TCGA data analyzed for DEK expression in esophageal squamous cell carcinomas (ESCC) versus normal tissue and (B) in adenoma compared to squamous cell carcinoma (SCC). Analysis was performed using RNAseq (V2) and clinical (histological subtype) data for esophageal carcinoma (ESCA) and was downloaded from GDAC Firehose.
(EPS)
